# Vertical Diamond p-n Junction Diode with Step Edge Termination Structure Designed by Simulation

**DOI:** 10.3390/mi14091667

**Published:** 2023-08-26

**Authors:** Guangshuo Cai, Caoyuan Mu, Jiaosheng Li, Liuan Li, Shaoheng Cheng, Qiliang Wang, Xiaobiao Han

**Affiliations:** 1 School of Optoelectronic Engineering, Guangdong Polytechnic Normal University, Guangzhou 510665, China; 2 State Key Laboratory of Superhard Materials, College of Physics, Jilin University, Changchun 130012, China; 3 Industry-Education-Research Institute of Advanced Materials and Technology for Integrated Circuits, Anhui University, Hefei 230093, China

**Keywords:** diamond, p-n junction diode, junction terminal extension, simulation

## Abstract

In this paper, diamond-based vertical p-n junction diodes with step edge termination are investigated using a Silvaco simulation (Version 5.0.10.R). Compared with the conventional p-n junction diode without termination, the step edge termination shows weak influences on the forward characteristics and helps to suppress the electric field crowding. However, the breakdown voltage of the diode with simple step edge termination is still lower than that of the ideal parallel-plane one. To further enhance the breakdown voltage, we combine a p-n junction-based junction termination extension on the step edge termination. After optimizing the structure parameters of the device, the depletion regions formed by the junction termination extension overlap with that of the p-n junction on the top mesa, resulting in a more uniform electric field distribution and higher device performance.

## 1. Introduction

Diamond-based devices are promising for high-frequency and high-power applications and can be worked under harsh conditions (high temperature, strong radiation, etc.) [1,2,3]. Diamond is an indirect bandgap semiconductor material with a bandgap of about 5.5 eV, thermal conductivity as high as 22 W/(cm·K), and room temperature electron and hole mobility as high as 4500 cm^2^/(Vs) and 3380 cm^2^/(Vs), which is much higher than that of the third-generation semiconductor materials GaN and SiC. A diamond diode presents a wide range of applications for the high-power electronic devices and high-frequency, high-power microwave devices [4]. In addition, due to the large exciton binding energy (80 meV), a diamond diode can realize high-intensity, free-exciton emission (wavelength of about 235 nm) at room temperature, which has a greater potential in the deep ultraviolet and the extreme UV deep-ultraviolet detectors [5]. Compared with the unipolar Schottky barrier diodes (SBDs) [6], the bipolar p-n diodes (PNDs) are attractive due to their capability of handling higher currents and high voltages at the same time [7,8,9,10,11]. In addition, they present lower on-resistance (*R_on_*) and less static power loss in high-voltage applications thanks to the conductivity modulation effect [12]. However, a maximum electric field exists at the interface between the n-diamond cathode and p-diamond drift layers. Especially, the depletion layer expands both vertically and laterally at the junction edge, resulting in electric field crowding and premature breakdown. Generally, edge-termination (ET) structures are strongly required to suppress the electric field crowding to maximize the material properties of the diamond.

For the PNDs, various advanced ET techniques have now been adopted to alleviate electric field crowding, including field plates (FPs), guard rings (GRs), junction termination extension (JTE), and ion implantation, as well as beveled/step mesa [13,14,15,16]. On the one hand, FP termination shows a high density of defects at the dielectric/diamond interface because there is no native oxide for diamond [17] which degrades the long-term reliability of the device. On the other hand, although the GRs and JTE terminations can be fabricated along with the PND process [18,19], they require precise design and take a relatively larger area. Mesa structure is widely used in GaN and SiC PNDs because of its simple fabrication process [20,21]. After etching the drift layer, the lateral expansion of the depletion layer around the electrode edge can be suppressed. Fukushima et al. reported that a deeply and vertically etched mesa structure is beneficial in fabricating vertical GaN PNDs with a low leakage current and avalanche capability [22]. The device simulation demonstrates that the electric field crowding is completely suppressed when the mesa is deeper than the depletion region, resulting in a uniform electric field distribution. Ohta et al. also reduced the electric field concentration of the mesa sidewall successfully for GaN PND by applying a simple two-step mesa structure [23]. Excellent avalanche capabilities without sacrificing any forward characteristics are realized at a high breakdown voltage (*V_BD_*) of 4.8 kV [23,24,25].

Generally, it is not easy to etch diamond deeply due to its high hardness and high atomic density. Therefore, a multi-step structure with shallow steps is regarded as more suitable for diamond-based diodes. However, the development of diamond PNDs with a mesa structure is rarely reported. Herein, we perform a comprehensive optimization of a mesa-based termination structure for diamond PNDs by using the Silvaco technology computer-aided design (TCAD) software (Version 5.0.10.R). A combined structure of step mesa and JTE is proposed to balance the electric performance and device fabrication. This work can provide a reference for the future development of high-power and high-voltage diamond bipolar power devices.

## 2. Design Models and Calibration

**Figure 1:**
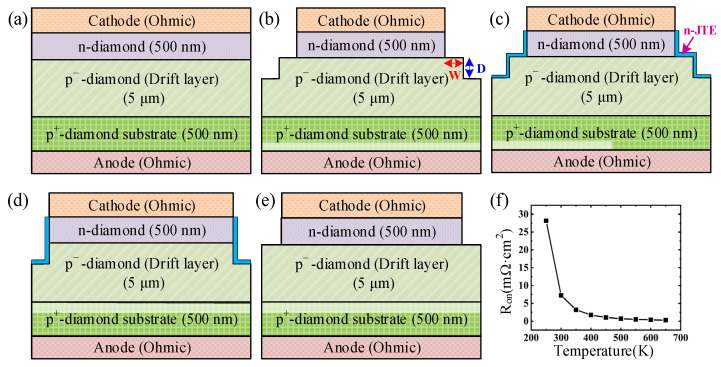
Schematic diagram of (**a**) ideal p-n junction, (**b**) step ET, (**c**) step ET with JTE structure, (**d**) deeply etched ET with JTE structure, and (**e**) conventional PND. (**f**) is the typical resistance versus temperature.

The electric performances of diamond PNDs were simulated by the commercial Silvaco TCAD software. Four kinds of diamond PNDs with different ET structures were designed for comparison (Figure 1). All the devices possess a similar structure consisting of 500 nm of heavily doped p^+^-diamond, 5 μm of a lightly doped p^−^-diamond drift layer, and 500 nm of a n-type doped diamond from bottom to top of the surface. The carrier concentrations of p^+^, p^−^, and n-type diamond were set to 2 × 10^20^ cm^−3^, 1.5 × 10^15^ cm^−3^, and 2.5 × 10^20^ cm^−3^, respectively. Figure 1a shows the ideal parallel-plane PND (the lengths of the p-type and n-type layers are the same), in which the *V_BD_* depends only on the thickness of the p^−^-diamond drift layer. The PND with a simple step ET structure is shown in Figure 1b, in which width (W) and depth (D) represent the interval of the n-diamond/mesa edge and the etching depth, respectively. As reported in our previous work [18], the beveled p-n JTE is an effective method for suppressing the electric field crowding. Therefore, we also propose a step ET with a JTE structure (Figure 1c) and a deeply etched ET with a JTE structure (Figure 1d) for evaluation. All the electrical performances of the devices with different TE structures are compared with that of a conventional PND (the n-type layer is partially etched with the metal electrode as a mask, Figure 1e).

During the TCAD simulation, a common phonon-assisted tunneling model, the Parallel-Electric-Field-dependent mobility model, Selberherr’s ionization model, and the incomplete ionization model were added [22]. The activation energy of the acceptor and donor are set to 0.36 eV and 0.57 eV, respectively [26]. Generally, it requires a relatively high temperature to effectively ionize the dopants and realize a small resistance due to the large activation energy. Therefore, we first evaluate the typical variation of resistance versus temperature (shown in Figure 1f) with the doping concentrations set as 1.5 × 10^15^ cm^−3^, 2 × 10^20^ cm^−3^, and 2.5 × 10^20^ cm^−3^ for the p^−^, p^+^, and n^+^ layers, respectively. When the temperature increases from 250 K to 650 K, the resistance rapidly decreases and nearly remains constantly higher than 500 K. Considering the actual working conditions, we think that 550 K is the suitable temperature used for simulation to obtain small resistance. The current-voltage (*I-V*) curve is used to calculate the current density, turn-on voltage (*V_on_*, extracted by linear fitting the forward region of the *I-V* curve), and *R_on_* (deduced by the differentiation of the *I-V* curve), while the electric field distribution is used to determine the *V_BD_*. It is worth noting that the critical breakdown electric field for diamond is set to be 6 MV/cm. The reason is that the ionization coefficient values are different in many previous works due to the immaturity of the diamond material and device [27].

## 3. Results and Discussion

**Figure 2:**
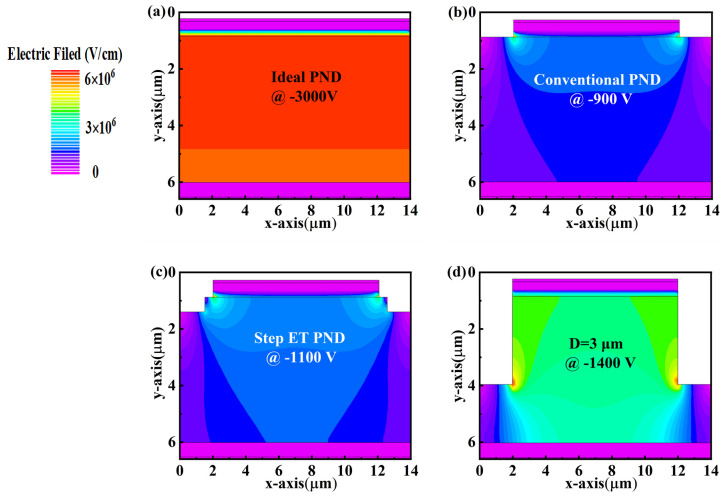
Electric field distribution of (**a**) ideal p-n junction device, (**b**) conventional p-n junction device, (**c**) step ET, and (**d**) deeply etched device with an etching depth of 3 μm when a reverse voltage is applied.

Firstly, we simulated the electrical performance of the diamond PNDs with different termination structures. Figure 2 shows the electric field distributions at the *V_BD_* of the PNDs. It demonstrates that the ideal diamond PND presents a high *V_BD_* of approximately 3000 V (as shown in Figure 2a) because the same length of the p-type and n-type layer eliminate the edge. On the other hand, The *V_BD_* of the conventional diamond PND without ET is only approximately 900 V, showing two obvious electric field crowding positions local at the edge of the n-type layer (Figure 2b). Therefore, two structures of shallow step mesa and deeply etched mesa are designed to relieve the electric field crowding at the p-n junction. As shown in Figure 2c, part of the electric field crowding at the edge of the p-n junction can be released by the step with a (W, D) of (0.5, 0.5), which enhances the *V_BD_* effectively to approximately 1100 V. Observed from Figure 2d, the deeply etched mesa (with a depth of 3 μm) shifts the crowding points to the mesa bottom and generates a much more uniform electric field distribution in the drift layer, resulting in the undoubtedly improved *V_BD_* to approximately 1400 V. It is worth noting that the variation in the termination structure shows weak influences on the forward characteristics of the PNDs (not shown), presenting a comparable *V_on_* of 5.4 V and *R_on_* of approximately 0.28 mΩ·cm^2^. It is worth noting that the device is fully turned on when the forward voltage is 10 V and the *R_on_* is relatively stable. The corresponding Baliga’s figure of merit (BFOM = *V_BD_* ^2^/*R_on_*) values are 30.0, 2.9, 2.42, and 6.24 GW/cm^2^ for the ideal PND, conventional PND, step mesa PND, and deeply etched mesa PND, respectively. It demonstrates that the deeply etched mesa is a more effective termination structure than the step mesa. However, it is not easy to etch diamond deeply due to its high hardness and high atomic density. Taking into account the device performance and the fabrication process, the step mesa with a relatively smaller etching depth in each process is chosen for further evaluation. This is almost in agreement with the values of Von, Ron, and BFOM reported by Traoré et al. [11]. Therefore, we consider the simulation results to be more credible.

**Figure 3:**
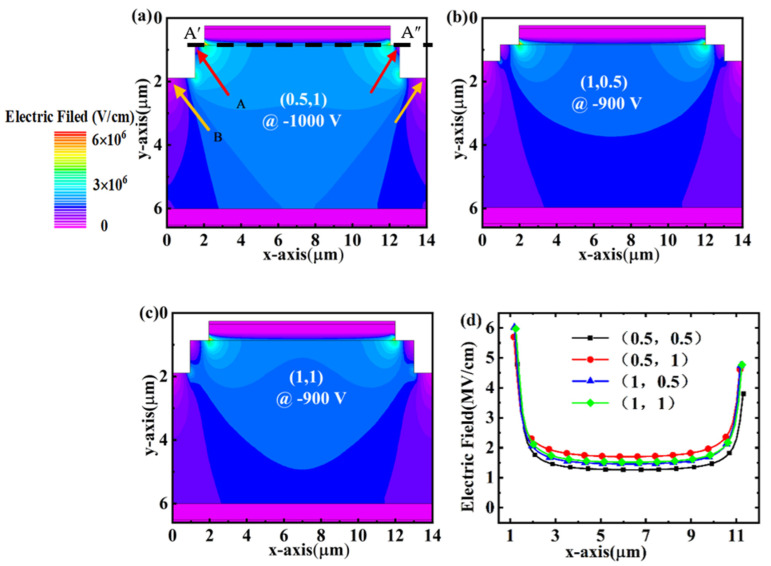
Electric field distributions under *V_BD_* for the PNDs with step parameters of (**a**) (0.5, 1), (**b**) (1, 0.5), and (**c**) (1, 1), respectively. (**d**) The electric field profiles at 900 V extracted along the maximum electric field (cutline A′A″).

**Figure 4:**
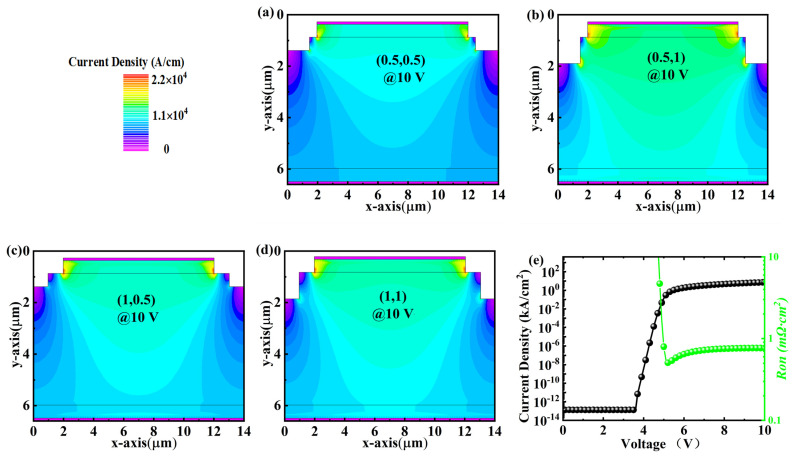
Current density distributions under 10 V for the PNDs with step parameters of (**a**) (0.5, 0.5), (**b**) (0.5, 1.0), (**c**) (1.0, 0.5), and (**d**) (1.0, 1.0), respectively. (**e**) is the typical current-voltage and *R_on_* of the device with a (W, D) of (0.5, 0.5).

The dimensions of W and D are the key parameters for the PND devices with a two-step mesa structure. In this section, four sets of W and D are adopted as (0.5, 0.5), (0.5, 1), (1, 0.5), and (1, 1), respectively. As summarized in Table 1, the PND with a (W, D) of (0.5, 0.5) shows the best *V_BD_* of 1100 V, while the increase in W or D will decrease the *V_BD_*. By comparing the electric field distributions at the *V_BD_* in Figure 2c and Figure 3a, the drift layer locations beneath the step become thin (marked as region B by the orange arrow) when the D increases to 1 μm, resulting in the degradation in *V_BD_*. The variation of *V_BD_* is consistent with the electric field profile along the maximum electric field. When the reverse voltage is 900 V, the device’s electric field distribution is basically the same, both at the edge of the p-n junction where the electric field is concentrated. The difference is that the device electric field is obviously effectively dispersed when W, D is 0.5, 0.5, and, thus, the (0.5, 0.5) BV is relatively high (Figure 3d). Similarly, we can also observe that the electric field distribution disappears in the drift layer around the step corner (marked as region A by the red arrow) when the W increases to 1 μm. However, the variation in W and D shows slight influences on the forward characteristics of the PNDs (Table 1, Figure 4e), presenting a comparable *V_on_* of 5.4 V and *R_on_* of approximately 0.47 mΩ·cm^2^. Figure 4a–d show the current density distribution at a forward voltage of 10 V of PNDs with (W, D) of (0.5, 0.5), (0.5, 1), (1, 0.5), and (1, 1), respectively. The results show that the current distributions are comparable to each other, which also can be confirmed by the *I-V* curves in Figure 4e,f. The corresponding BFOM values are 2.42, 2.12, 1.75, and 1.74 GW/cm^2^ for the (0.5, 0.5), (0.5, 1), (1, 0.5), and (1, 1), respectively. Therefore, the relatively smaller W and D are beneficial to provide a wider and more uniform electric field distribution in the drift layer for the PND with the step mesa. It can be seen that the reverse BV and BFOM values of the device when using (W, D) of (0.5, 0.5) are close to the 1550 V, 2.64 GW/cm^2^ reported by Lin et al. [22]. This also demonstrates that the stepped countertop can alleviate the electric field crowding at the p-n junction.

**Figure 5:**
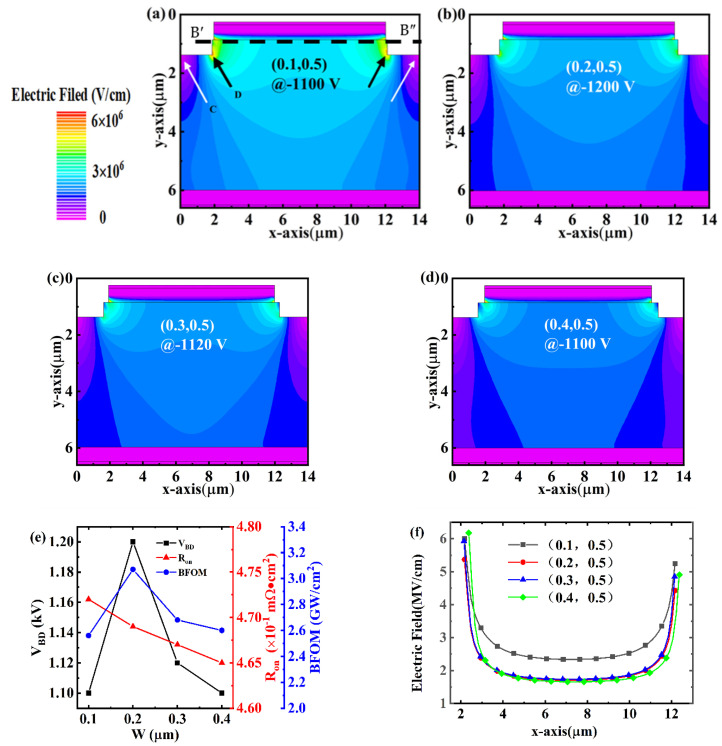
Electric field distributions under *V_BD_* for the PNDs with step parameters (W, D) of (**a**) (0.1, 0.5), (**b**), (0.2, 0.5), (**c**) (0.3, 0.5), and (**d**) (0.4, 0.5), respectively. (**e**) is the plot of *V_BD_* and *R_on_* as a function of W. (**f**) is the electric field profiles at 1100 V extracted along the maximum of electric field (cutline B′B″).

**Figure 6:**
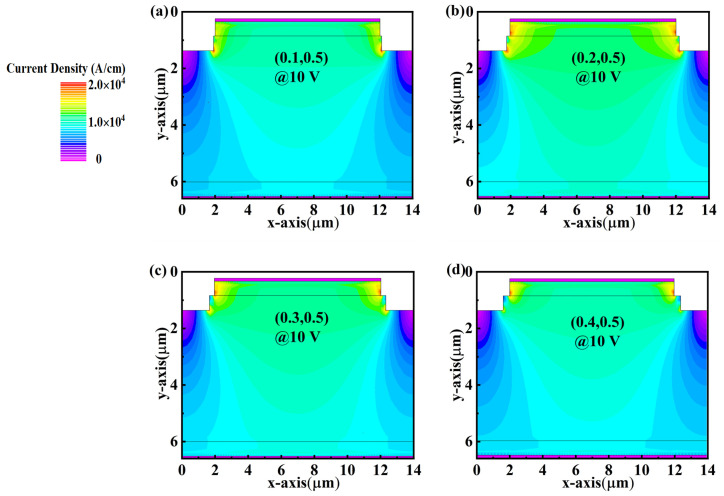
Current density distributions under 10 V for the PNDs with step parameters of (**a**) (0.1, 0.5), (**b**) (0.2, 0.5), (**c**) (0.3, 0.5), and (**d**) (0.4, 0.5), respectively.

As summarized in Table 1, we found that the variation of *V_BD_* versus W is more obvious than that versus D. Therefore, we keep the D and adopt four sets of Ws as (0.1, 0.5), (0.2, 0.5), (0.3, 0.5), and (0.4, 0.5) for further optimizations. Figure 5 shows the electric field distributions under *V_BD_* as well as the corresponding *V_BD_* and *R_on_* for the PNDs. It is found that the drift layer locals beneath the step (marked as region C by the white arrow) contribute to sustaining the reverse bias, but the electric field distribution tends to crowd around the bottom corner (marked as region D by the black arrow) of the step when the W is 0.1 μm. In the reverse voltage of 1100 V, the breakdown location is due to the different devices and leads to a slight difference in the distribution of the electric field. When W = 0.1 μm, the device breakdown location for the right-angle region is close, so the size of the step under result did not play a role in mitigating the role of the electric field density, resulting in a relatively low BV. When W is more than 0.2 μm, the step away edge of the p-n junction is relatively far, resulting in the weak dispersion of the electric field ability. Therefore, the BV decreases. When W increases to 0.2 μm, the electric field distributions around the p-n junction interface and the bottom corner become more uniform, resulting in the suppression of the crowding point and reaching a maximum *V_BD_* of 1200 V (Figure 5f). However, the electric field distribution in region D (by the black arrow) tends to disappear when W is further increased to 0.3 and 0.4 μm. On the other hand, the variation in W shows slight influences on the forward characteristics of the PNDs (Figure 6a–d), presenting a comparable *V_on_* of 5.4 V and *R_on_* of approximately 0.47 mΩ·cm^2^. The corresponding BFOM values are 2.56, 3.07, 2.68, and 2.60 GW/cm^2^ for the (0.1, 0.5), (0.2, 0.5), (0.3, 0.5), and (0.4, 0.5), respectively. It can be seen that when the (W, D) of the device is (0.2, 0.5), the BFOM of the device is the maximum, but it is only 10% of the ideal device, which makes it difficult to be commercially applied.

**Figure 7:**
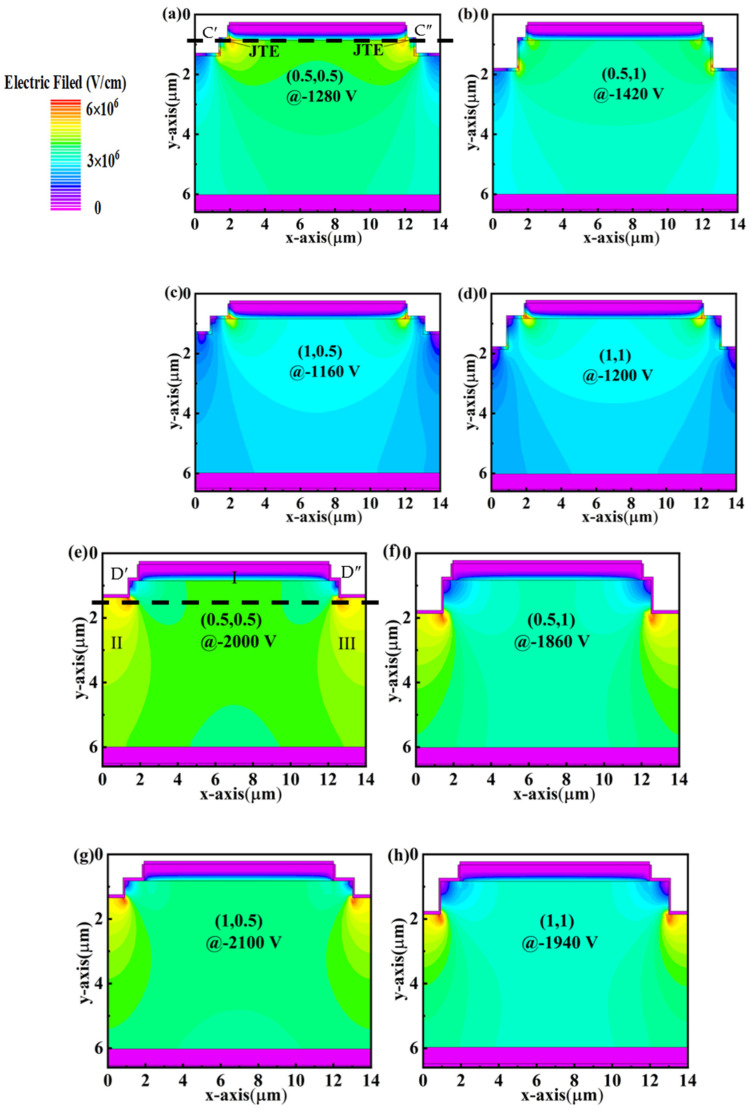

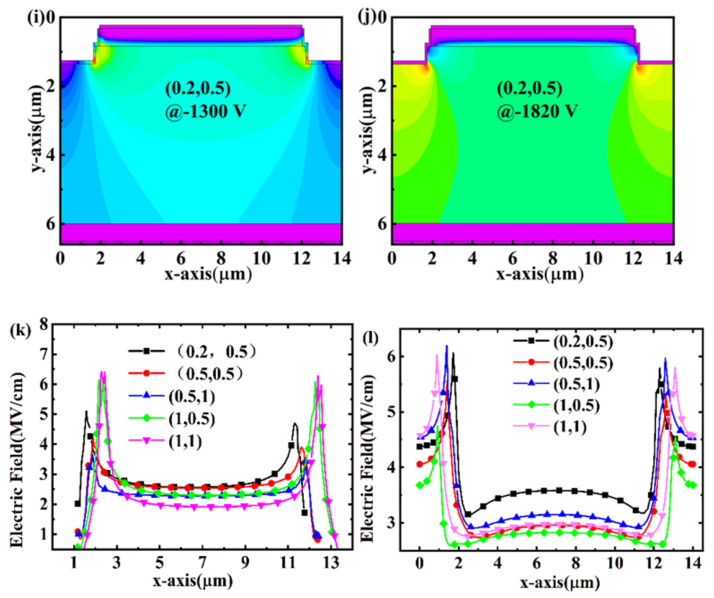
Simulated electric field distributions of the step ET PNDs with different (W, D) values. (**a**–**d**) are the electric field distributions with the JTE doping concentration of 3 × 10^18^ cm^−3^; (**e**–**h**) are the electric field distributions with the JTE doping concentration of 2 × 10^22^ cm^−3^. (**i**,**j**) are the electric field distributions of (0.2, 0.5) with the JTE doping concentration of 3 × 10^18^ cm^−3^ and 2 × 10^22^ cm^−3^, respectively. (**k**,**l**) are the electric field profiles in low and high JTE concentrations at 1200 V or 1900 V extracted along the maximum electric field (cutline C′C″ and D′D″), respectively.

Based on the discussion above, although the fabrication of the step mesa is relatively easier, the *V_BD_* value is far away from the theoretical one. Previous works report that the JTE structure with a proper doping concentration also can effectively reduce the electric field crowding and enhance the *V_BD_* of the device [16]. Therefore, we simulated the hybrid structure of the step mesa and JTE on the device performances with different JTE doping concentrations. Herein, we only present two typical doping concentrations, namely a low concentration of 3 × 10^18^ cm^−3^ and an optimal concentration of 2 × 10^22^ cm^−3^, to reveal the variation of the electrical properties. It is worth noting that the JTE on the mesa presents a relatively smaller device area compared with the conventional planar JTE. As shown in Figure 7, the electric field distributions are similar to the PNDs without JTE when the concentration of JTE is below 3 × 10^18^ cm^−3^. The possible reason is attributed to that the depletion effect of the JTE is much weaker than that of the p-n junction. However, the JTE can introduce an extra depletion region around the step bottom to expand the electric field distribution in the drift layer locals beneath the step bottom. Then, all the PNDs with different (W, D) show a slightly enhanced *V_BD_* compared with the PNDs that only possess a step mesa (as summarized in Table 2). When the concentration of JTE reaches 2 × 10^22^ cm^−3^, the depletion regions formed by the JTE obviously adjust the electric field distribution.

The electric field distribution of PNDs can be divided into three regions and labeled into Ⅰ, Ⅱ, and Ⅲ (Figure 7e), which is contributed by the p-n junction, the left JTE, and the right JTE, respectively. A relatively large depth decreases the effective distance between cathode and anode electrodes, resulting in enhanced crowding and decreased *V_BD_* (Figure 7f). In addition, we also found that a relatively large W is beneficial to arrange the electric field distribution introduced by the p-n junction on the top mesa and by the JTE around the bottom mesa, resulting in a more uniform electric field distribution and higher *V_BD_* (Figure 7h). When using a JTE with a doping concentration of 3 × 10^18^ cm^−3^, it can be seen from the electric field distribution that the devices with a W, D of (0.5, 0.5) have a stronger ability to mitigate the electric field concentration effect and are significantly lower than the devices with (0.2, 0.5) and (0.5, 1). When using a JTE with a doping concentration of 2 × 10^22^ cm^−3^, it can be seen from the electric field distribution that the devices with a W, D of (1, 0.5) have a stronger ability to mitigate the electric field concentration effect and are significantly lower than the devices with (0.5, 1). The electric field concentration effect mitigation is stronger and significantly lower than the devices with (0.5, 1) and (1, 1) (Figure 7k,l). Combining the effects of both W and D, a maximum *V_BD_* of 2100 V is realized for the PND with a (W, D) of (1, 0.5), step mesa, and JTE (Figure 7g). On the other hand, the current distributions (not shown) are nearly the same for the PNDs with different (W, D) values, as summarized in Table 2. It is worth noting that the (W, D) of (0.2, 0.5) is the optimum structure for the PNDs without JTE. We also provide the electric field distributions for a (W, D) of (0.2, 0.5) with JTE doping concentrations at 3 × 10^18^ cm^−3^ and 2 × 10^22^ cm^−3^ (Figure 7i,j) for comparison. As shown in the figures, the *V_BD_* is smaller than the (W, D) of (0.5, 1) structure after the introduction of JTE.

**Figure 8:**
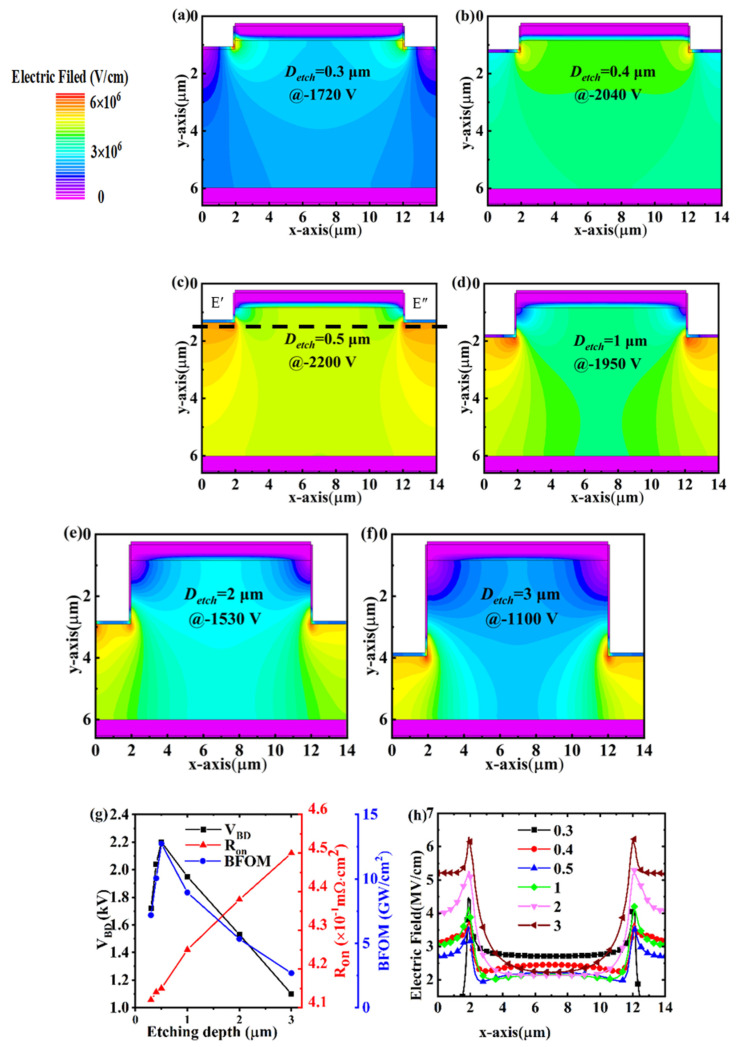
Electric field distribution of deeply etched PNDs at a reverse voltage of *V_BD_* with (**a**) D = 0.3 μm; (**b**) D = 0.4 μm; (**c**) D = 0.5 μm; (**d**) D = 1 μm; (**e**) D = 2 μm; (**f**) D = 3 μm, respectively. (**g**) is the deduced *R_on_* and *V_BD_* plotted versus etching depth. (**h**) is the electric field profiles at 1100 V extracted along the maximum electric field (cutline E′E″).

For comparison, we also evaluate the effect of JTE on the deeply etched mesa structures. The simulated electric field distributions under the *V_BD_* for the PNDs with different etching depths are shown in Figure 8. It is observed that the electric field is distributed at the interfaces of the JTE and p-n junction. When the etching depth is relatively small or large, the electric field tends to crowd around the JTE interface, especially the corner of the trench. On the other hand, the electric fields at the interfaces of the JTE and p-n junction merge together, resulting in the highest *V_BD_*, a maximum *V_BD_* of 2300 V (D = 0.5 μm). This value is comparable to that of the step device with (1, 0.5). Most of the electric field distribution shifts downward to the ohmic contact electrode when the etching depth is increased. Furthermore, the drift region that does not sustain the reverse bias becomes broad around the p-n junction interface with an increasing depth. Therefore, the degradation of *V_BD_* is ascribed to the suppressed effective drift layer thickness that sustains the bias. Also, we observe that the forward conduction current of the device decreases slightly with the increasing etching depth, which is attributed to the gradually suppressed conduction path. It can be seen that as the depth of etching increases, the electric field at the corners of the device gradually concentrates with a fixed reverse withstand voltage of 1100 V, which leads to a decrease in the withstand voltage performance (Figure 8h). Therefore, we found that the deeply etched mesa is not required for the step edge termination with the assistance of the JTE.

From Table 3, it can be seen that the profoundly etched device using the JTE has the best reverse voltage withstand performance among the currently known diamond diode devices, which provides an idea for the future development of diamond power diode devices.

## 4. Conclusions

In summary, a step ET with a JTE structure is designed for vertical diamond p-n junction diodes using TCAD simulation. Firstly, a simple step structure is used to mitigate the electric field crowding at the p-n junction. It can modify a certain amount of electric field distribution and increase the device breakdown voltage. At the same time, there is no significant change in the *R_on_* and forward current under the forward voltage. After that, the performance of the step with different structure parameters is explored. It is found that part of the drift layer will not sustain the reverse bias with the increase in W or D, resulting in the degradation in *V_BD_*. However, the breakdown voltage is still far lower than that of the ideal parallel-plane one.

On the other hand, we propose a novel structure to improve the voltage resistance by combining the JTE and step. The electric field distributions are similar with the PNDs without ET when the concentration of JTE is lower than that of the n-diamond layer, which is attributed to that the depletion effect of the JTE being much weaker than that of the p-n junction. When the concentration of JTE reaches 2 × 10^22^ cm^−3^, the depletion regions formed by the JTE obviously adjust the electric field distribution. For the PND with a (W, D) of (1, 0.5) step mesa, the electric field distributions introduced by the p-n junction on the top mesa and the JTE around the bottom mesa overlap each other, resulting in a more uniform electric field distribution and higher *V_BD_*. Finally, a device with a *R_on_* of 0.415 mΩ·cm^2^, *V_BD_* of 2200 V, and BFOM of 12.74 GW/cm^2^ is obtained.

## Figures and Tables

**Table 1:** Simulated electrical characteristics for the SBDs with different W and D.

(W, D) (μm/μm)	*V_BD_* (V)	*Von* (V)	*R_on_* (mΩ·cm^2^)	BFOM (GW/cm^2^)
(0.5, 0.5)	1100	5.4	0.5	2.42
(0.5, 1)	1000	5.4	0.471	2.12
(1, 0.5)	900	5.4	0.461	1.75
(1, 1)	900	5.4	0.464	1.74

**Table 2:** Simulated electrical characteristics for PNDs with different ET structures and parameters.

(W, D) (μm/μm)	JTE (cm^−3^)	*V_BD_* (V)	*V_on_* (V)	*R_on_* (mΩ·cm^2^)	BFOM (GW/cm^2^)
(0.2 ,0.5)	3 × 10^18^	1300	5.4	0.463	3.93
(0.5, 0.5)	3 × 10^18^	1280	5.4	0.460	3.56
(0.5, 1)	3 × 10^18^	1420	5.4	0.464	4.34
(1, 0.5)	3 × 10^18^	1160	5.4	0.461	2.91
(1,1 )	3 × 10^18^	1200	5.4	0.461	3.12
(0.2, 0.5)	2 × 10^22^	2010	5.5	0.418	7.92
(0.5, 0.5)	2 × 10^22^	2000	5.5	0.413	9.68
(0.5, 1)	2 × 10^22^	1860	5.5	0.418	8.27
(1, 0.5)	2 × 10^22^	2100	5.5	0.411	12.87
(1, 1)	2 × 10^22^	1940	5.5	0.454	8.28

**Table 3:** Comparison of pressure resistance of different structures.

Diode Type	p-Layer (μm)	Von (V)	Ron (mΩ·cm^2^)	BV (V)	Refs.
SBD	4.5	−0.5	135	560	[28]
SBD	1.3	3	0.006	1000	[29]
JTE SBD	4	2.6	0.91	1550	[16]
metal–insulator–p+(MIP) SBD	18	-	-	1100	[30]
PN diode	3.5	2.6	0.97	1350	[22]
PN diode	3	5	0.47	600	[11]
PN diode	5	5.4	0.411	2200	This work

## Data Availability

Data available on request due to restrictions eg privacy or ethical. The data presented in this study are available on request from the corresponding author.
